# Occupational Therapy in Psychiatric Short-Term Hospitalization Units: Scoping Review

**DOI:** 10.1007/s10597-024-01286-3

**Published:** 2024-05-09

**Authors:** Uxia García-Gestal, Miguel-Ángel Talavera-Valverde, Ana-Isabel Souto-Gómez

**Affiliations:** 1 Centro Ocupacional Pascual Veiga, A Coruña, Spain; 2 PhD. Health Science Department, Faculty of Health Science, Universidade da Coruña, Campus de A Coruña, A Coruña, Spain; 3 Area Sanitaria Ferrol, A Coruña, Spain; 4 Integra Saúde Research Unit, Universidade da Coruña, A Coruña, Spain; 5 Department of Social Work, Escola de Traballo Social, Universidad de Santiago de Compostela, A Coruña, Spain

**Keywords:** Occupational Therapy, Mental Health, Hospitals, Psychiatric, Day Care, Medical

## Abstract

Given the context, the overarching aim is to identify scientific publications on occupational therapy in Psychiatric-Short-Term-Hospitalization-Units. Specific objectives include: (a) Analyzing the historical development of this research area; (b) Synthesizing existing evidence on the nature of documentary sources on occupational therapy in in Psychiatric-Short-Term-Hospitalization-Units; (c) Detailing the volume of scientific literature on occupational therapy in these units; (e) Evaluating available evidence on occupational therapy interventions to improve functionality, quality of life, and recovery in individuals admitted to in Psychiatric-Short-Term-Hospitalization-Units. A scoping review method was employed to conduct a historical mapping of research on in Psychiatric-Short-Term-Hospitalization-Units. The review proceeded in five stages following PRISMA guidelines. After applying selection criteria, the search identified 446 references. Findings are presented under three headings: (a) Historical trends in the scientific literature on occupational therapy and in Psychiatric-Short-Term-Hospitalization-Units; (b) Nature and volume of articles included in the occupational therapy synthesis in Psychiatric Short-Term Hospitalization Units; (c) Data extraction on methodological variables in the research of occupational therapy articles in in Psychiatric-Short-Term-Hospitalization-Units; and (d) Data extraction on research outcome variables of occupational therapy articles in Psychiatric Short-Term Hospitalization Units. The growth of occupational therapy in in Psychiatric-Short-Term-Hospitalization-Units is emphasized, with an increase in qualitative studies. Occupational therapy is underscored as an integral part of care, supporting the diversity and effectiveness of interventions. Common diagnoses include schizophrenia and depressive disorders. Group interventions and the spiritual dimension positively influence the quality of care and meaningful routines for recovery in in Psychiatric-Short-Term-Hospitalization-Units.

Health, according to the Organización Mundial de la Saludn (OMS) (2014), is not merely the absence of disease but a comprehensive state of physical, mental, and social well-being, including factors like quality of life, bodily function, and emotional well-being. In fact, *the significance of occupation*, as emphasized by Harmer and Orrell (2008), plays a vital role in promoting well-being through purposeful activities such as work, leisure, and self-care, which vary for each individual. This close relationship between health and occupation underscores the significant impact activities have on both mental and physical health.

Hence, occupational therapy, as substantiated by research from Schuch et al. (2016) and Eakman (2014), acknowledges the beneficial effects of meaningful activities on health, well-being, and overall life satisfaction. It serves as a valuable discipline in improving individuals' health and well-being by tackling occupational challenges that hinder their engagement in meaningful activities. This approach is in harmony with the holistic view of health, considering it as encompassing physical, mental, and social well-being.

## The Importance of Occupational Therapy in the Field of Mental Health

Occupational therapy, focusing on human occupation and well-being (AOTA, 2020; WFOT, 2017), is crucial in mental health, enhancing occupational competence and quality of life for individuals facing challenges. -Moruno-Miralles and Talavera-Valverde (2011) stress its role in fostering autonomy and independence for those with mental health issues, enabling active engagement and finding purpose in life. D'Amico et al. (2018) highlight its ability to enhance occupational performance and life satisfaction, while Gibson et al. (2011) affirm its effectiveness in addressing mental health challenges and improving daily functioning. In summary, occupational therapy is invaluable in maintaining competence, promoting health, enhancing well-being, and preventing relapses, directly impacting individuals' health (Moruno-Miralles & Talavera-Valverde, 2011).

## Occupational Therapy in the Context of Psychiatric Short-Stay Hospitalisation Units

Occupational therapy holds a pivotal role within Psychiatric Short-Stay Hospitalisation Units (UHP), concentrating on enhancing well-being and optimizing occupational performance for overall health improvement. Moruno-Miralles and Talavera-Valverde (2011) stress its capacity to empower service users to develop occupational competence, facilitating autonomous and fulfilling engagement in daily activities, thus supporting their recovery and community reintegration.

Noyes and Griffin (2019) highlight how occupational therapy aids in reacquiring daily life skills, fostering independence, and bolstering patients' self-esteem within UHP settings.

Additionally, occupational therapy fosters participation skills and social engagement, particularly beneficial for those confronting social isolation or interpersonal challenges (Haracz & Ashby, 2019). Brown et al. (2019) affirm its role in assisting users to identify and cultivate meaningful interests and activities, positively influencing their psychological and emotional well-being. Furthermore, occupational therapy contributes significantly to discharge planning, aiding users in accessing community resources and devising transition plans (Holm & Mu, 2012).

Even from a more biomedical perspective, Rocamora-Montenegro et al. (2021) confirm that this discipline promotes the acquisition of skills to manage stress and anxiety, as well as learning relaxation techniques and emotional control.

## Justification

In the current era, with a heightened focus on mental health, attention has expanded to encompass occupational therapy. However, despite its increasing relevance, a comprehensive review of the current state of knowledge and research gaps in this area has yet to be undertaken. As a result, there is no precise definition of the scientific evidence supporting occupational therapy practice in UHP, unlike other disciplines such as nursing (Doedens et al., 2020), medicine (Gaynes et al., 2017), or psychology (Evlat et al., 2021; Jacobsen et al., 2018).

Consequently, conducting a review of occupational therapy in UHP is essential for improving the quality of psychiatric care. Such a review will pinpoint opportunities to enhance the effectiveness and efficiency of occupational therapy, ultimately generating robust scientific evidence to inform decision-making processes.

## Objectives

Given the above, the overarching objective is to identify scientific publications on occupational therapy in UHP. Specific objectives include: (a) Analyzing the historical development of this research area; (b) Synthesizing existing evidence on the nature of documentary sources on occupational therapy in UHP; (c) Detailing the volume of scientific literature on occupational therapy in these units; (e) Evaluating available evidence on occupational therapy interventions to improve functionality, quality of life, and recovery in individuals admitted to UHP.

## Methodology

### Study Type

A scoping review was conducted following the PRISMA-SCR guidelines (Tricco et al., 2018), utilizing the methodological framework established by Arksey and O'Malley (2005) and further developed by Levac et al. (2010). This method aims to clarify the development of occupational therapy in UHP, capture and map a variety of evidence to illustrate the scope of the study area. The protocol was registered on the Open Science Framework before the start of the investigation.

### Research Question and Identification of Relevant Studies

Two research questions were established to guide the scoping review: (a) What is the nature and scope of the scientific literature/evidence on occupational therapy in UHP? and (b) How has occupational therapy research in UHP evolved over time?

A search was conducted in various databases (Ovid MEDLINE, PsycINFO, ProQuest ERIC, Web of Science -WOS-, CSIC, Dialnet, Pubmed Central, OTDBASE, and Scielo). Mesh terms (Hospitals, Psychiatric, Day Care, Medical, Mental Health, Occupational Therapy) were determined to facilitate/narrow the bibliographic search, and several keywords related to the review's topic were established (Psychiatric short-stay hospitalization units, Psychiatric short-stay units, Acute Mental units, short-stay crisis units, Short-stay mental health crisis units, Psychiatric inpatient units, Psychiatry, Occupational therapy, Ergotherapy, Occupational therapist, Ergotherapist, Hospitals, Psychiatric, Day Care, Medical, Mental Health).

Search strings were established using the primary matrix for the overall strategy: (occupational therap*) OR (ergotherap*) AND ((Psychiatric short-stay hospitalisation units) OR (Psychiatric short-stay units) OR (Acute Mental units) OR (short-stay crisis units) OR (Short-stay mental health crisis units) OR (psychiatric inpatient units)).

No year filter was applied, and the search for results was conducted until February 12, 2023.

### Study Selection

The identification and selection of relevant studies were guided by the following selection criteria:

- Inclusion Criteria: Peer-reviewed articles published in English, Spanish, and Portuguese addressing occupational therapy in UHP were included. There were no restrictions on the publication date.
- Exclusion Criteria: Conferences, theses, or opinion articles were excluded. Studies that did not assess occupational therapy in UHP were eliminated, as well as those focusing solely on occupational therapy in outpatient settings, studies concentrating on interventions other than occupational therapy in UHP, and studies not involving users or staff of UHP.

### Data Extraction

To facilitate the analysis of bibliographic references, search results from databases were stored in ZOTERO, a tool for organizing such references and documents. Duplicate articles were subsequently removed. Initially, documents were selected based on title relevance, excluding those lacking keywords or failing to meet inclusion criteria. A table was created for article selection, categorizing them by validity. Further selection rounds involved abstract review and, finally, full-text assessment, resulting in definitive documents meeting the established criteria.

The data from the definitive articles were extracted and incorporated into a data extraction table created using Excel v.16.26. The extraction process was independently performed by U.G.G. and A.I.S.G. Subsequently, M.A.T.V. reviewed the data extraction, with no disagreements identified during the review of titles, abstracts, and full texts. Furthermore, the mapping process was facilitated using Covidence Software to screen articles and conduct the full-text review.

### Classification and Data Analysis

The fourth stage involved organizing the data through an interactive process using three categories for grouping: (a) Scientific production variables; (b) Methodological variables; (c) Occupational therapy practice variables (Table 1).

**Table 1:** Variables analyzed in the study

Scientific production variable
Authorship
• Name: Authors' full names (last name, first name, middle name), listed alphabetically by last name if there are multiple authors
• Number of Authors: Total count of authors contributing to each article
• Country: Country of origin for each author
Scientific Journal
• Journal Name: Title of the journal where the article is published
• Year of Publication: The year when the article was initially published
• Language: Language utilized by the journal for its publications
• Journal Type Classifications: Indicates if the journal has an impact factor (JCR or SJR)
• Quartile: Evaluation indicator of a journal's significance relative to other journals in its field, classified as Q1, Q2, Q3, or Q4
Methodological Variables
• Methodology: Research methods for data collection in the articles, categorized as qualitative (involving words, narratives, or opinions), quantitative (comparing numerical data), or mixed (combining both)
• Study Design: Techniques or methods guiding the research process, such as experimental, phenomenological, descriptive, among others
• Study Population: Characteristics shared by participants in the study, including women, men, adolescents, individuals with mental health issues, users, professionals, among others
• Sex: Biological sex of the participants in the study
• Sample Size: Number of participants involved in the research
Occupational Therapy Practice Variables
• Device Characteristics: Setting or location where the research is conducted
• Medical Diagnoses: Assessment of individuals under study based on the Diagnostic and Statistical Manual of Mental Disorders-5th edition (DSM-V) criteria (American Psychiatric Association [APA], 2013)
• Types of Intervention: Actions implemented in the study
• Intervention Results: Outcomes of the intervention administered in the research
• Intervention Conclusions: Summary and reflection on the results and their implications

Our aim was to establish parameters for analyzing the literature to conduct a critical and comprehensive review. This stage involved a meticulous examination of the selected documentation, including reading and analyzing articles from indexed journals. Historical research trends were identified, commencing from the publication of the initial article in 1943 (Anonymous, 1943).

The results, or thematic variables, were categorized after theoretical saturation (Hernández-Sampieri & Mendoza, 2018) into four themes. Abductive reasoning, which combines deductive and inductive reasoning, was employed for this purpose, adapting the theoretical framework to empirical findings. Adjustments in the theoretical framework facilitated a conceptual interpretation of the data. This type of reasoning is common when exploring less-explored topics (Verd & Lozares, 2016).

Following Levac et al.'s (2010) qualitative content analysis approach, and aiming to (a) validate and enhance our understanding of findings, (b) seek feedback on coherence and direction of thematic analysis, and (c) facilitate knowledge transfer on occupational therapy's relevance in UHP, we conducted two consultations spaced approximately six months apart. The first involved experts—two professors and educators from Spanish universities, and two mental health professionals—recruited via snowball sampling. They were presented content blindly, unaware of each other's involvement. This initial consultation took place in July 2023, focusing on initial data and emerging categories. The second consultation occurred after completing data analysis.

Quantitative variables were expressed through frequency and percentage. Descriptive and inferential statistical analyses, utilizing the chi-square test, were conducted on different categories of scientific articles published between 1943 and 2023. Studies were grouped into 10-year periods for statistical comparison across phases.

We utilized the JBI Levels of Evidence developed by the Levels of Evidence and Grades of Recommendation Working Group of the Joanna Briggs Institute (JBI 2014) to assess the evidence (Effectiveness and Meaningfulness).

## Results

**Fig. 1:**
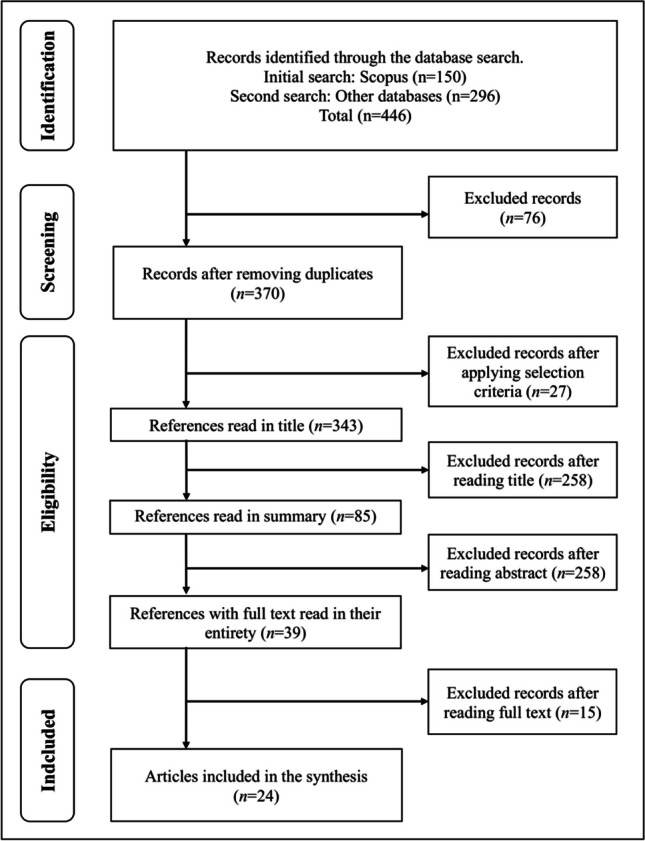
Diagram about or procedure for selecting ítems. Note. PRISMA Flow Diagram (Page et al., 2021)

The search strategies retrieved *n* = 446 references (*n* = 374 after removing duplicates). After applying the selection criteria, *n* = 24 references were identified (Fig. 1). Results are presented in 4 groups: (a) Historical trends in the scientific literature on UHP; (b) Nature and volume of publications on occupational therapy in UHP; (c) Data extraction on methodological variables in the research articles on occupational therapy in UHP; (d) Data extraction on outcome variables in research articles on occupational therapy in UHP.

### Historical Trends in the Scientific Literature on Occupational Therapy and UHP

**Fig. 2:**
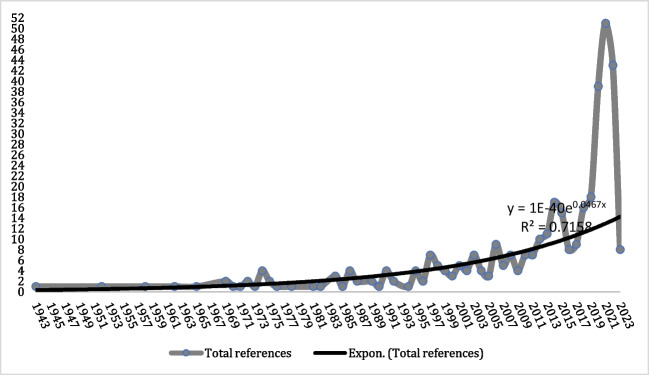
Literature on short-term psychiatric hospitalization since the first year of publication

The initial publication surfaced in 1943, marking the onset of a consistent uptrend in article publications over time (Fig. 2). Among the *n* = 374 documents retrieved, the majority, *n* = 369 (98.6%), comprised articles from indexed journals, including original studies and reviews. The remaining documents included book chapters, *n* = 3 (0.8%), doctoral theses, and conferences with *n* = 1 (0.3%) each.

To gauge the statistical significance of article publications over time, we organized them into ten-year intervals for comparative analysis. From 1943–1952 and 1953–1962, *n* = 2 articles were published in each period (0.6%). This count increased to *n* = 7 (1.9%) from 1963–1972, remaining at *n* = 11 (3%) from 1973–1982. The period from 1983–1992 saw *n* = 19 articles (5.1%), almost doubling to *n* = 35 (9.4%) from 1993–2002. There was a notable surge in the decade from 2003–2012, with *n* = 63 articles (16.9%), and a peak in 2013–2023, with *n* = 234 (62.7%). Statistical significance (*p* = 0.001) was observed solely in the periods 2003–2012 and 2013–2023 compared to other decades.

English is the dominant language in publications, comprising *n* = 326 (87.2%). It is trailed by Spanish with *n* = 14 (3.7%) and German with *n* = 13 (3.5%). Other languages (French, Arabic, Danish, Portuguese, Swedish, Chinese, Korean, Hebrew, Italian, or Polish) each represent less than 1% of publications, respectively.

### Nature and Volume of Articles Included in the Synthesis of Occupational Therapy in UHP

In the examination of the temporal distribution of publications, out of the total *n* = 446 papers on occupational therapy in UHP generated during the study duration, not all years meet the study's selection criteria. Among the *n* = 24 articles that did meet these criteria, the distribution was as follows: in 2014 and 2021, *n* = 3 (12.5%) articles were published each year. For the years 2006, 2016, 2017, and 2019, there were *n* = 2 (8.3%) articles each. Conversely, the years 1986, 1990, 1996, 2002, 2008, 2011, and 2020 each presented *n* = 1 (4.2%) article, respectively. It's noteworthy that when comparing the decades in which these *n* = 24 articles were published, no statistical significance (*p* = 0.001) was found to indicate one decade standing out from the others.

Taking into account the countries where the studies were conducted (Table 4), Spain has the highest representation with 29.2% (*n* = 7), followed by the United Kingdom with 20.8% (*n* = 5), the USA with 16.7% (*n* = 4), Australia with 12.5% (*n* = 3), and Canada with 8.3% (*n* = 2). Other countries (Iceland, Israel, and South Africa) each have figures of 4.1%  (*n* = 1) respectively.

Regarding languages, the analysis reveals a significant difference (*p* = 0.004) in the distribution of languages used in articles on occupational therapy in UHP, with English predominating at 70.8% (*n* = 17) compared to Spanish at 29.2% (*n* = 7). It's worth noting that the Canadian Journal of Occupational Therapy employs both English and French (Table 2).

**Table 2:** Number of articles published per journal and impact factor

Journal	Impact Indices and quartile		Language	Authors	Article	x̄
	JCR	SJR		n(%)	n(%)	
American Journal of Occupational	Q1	Q1	English	8(15.7)	4(16.7)	2
Archivos de la Memoria	N/A	N/A	Spanish	2(3.9)	1(4.2)	2
Australian Occupational Therapy Journal	Q3	Q1	English	5(9.8)	1(4.2)	5
British Journal of Occupational Therapy	Q4	Q2	English	9(17.6)	6(25)	2
Canadian Journal of Occupational Therapy	Q3	Q2	English/Francais	4(7.8)	2(8.3)	2
Indivisa	N/A	N/A	Spanish	2(3.9)	2(8.3)	1
Informaciones psiquiátricas	N/A	N/A	Spanish	1(2)	1(4.2)	1
Irish Journal of Occupational Therapy	N/A	Q3	English	4(7.8)	1(4.2)	4
Occupational Therapy in Health Care	Q3	Q3	English	4(7.8)	1(4.2)	4
Occupational Therapy in Mental Health	Q4	Q4	English	1(2)	1(4.2)	1
Revista Asociación Profesional Española de Terapeutas Ocupacionales	N/A	N/A	Spanish	3(5.9)	1(4.2)	3
South African Journal of Psychiatry	Q4	Q3	English	3(5.9)	1(4.2)	3
TOG (A Coruña)	N/A	N/A	Spanish	9(17.6)	2(8.3)	5
Total				51(100)	24(100)	2.3

Regarding journals and their impact factors, the 24 articles in this scoping review are distributed across 13 different peer-reviewed journals (Table 2). Among these, 53.8% (*n* = 7) are listed in both the Journal Citation Report and Scimago Journal Ranking. Notably, the latter includes one additional journal (Irish Journal of Occupational Therapy). The remaining journals are indexed in other databases with impact factors distinct from the aforementioned two. While there is a discernible contrast between journals included in JCR or SJR and those not, no statistical significance (*p* > 0.005) is evident when comparing them to journals absent from these indices. This discrepancy may arise by chance and might not signify a genuine association between impact factors and inclusion in JCR or SJR.

Similarly, in both JCR and SJR quartiles, Q3 journals prevail, constituting 23.1% (*n* = 3) each. Regarding author preferences for publication venues, two journals stand out: the British Journal of Occupational Therapy, with 25% (*n* = 6) of the articles, followed by the *American Journal of Occupational Therapy*, with 16% (*n* = 4). Other journals do not exceed 10% of the published articles.

Regarding authorship, a total of 24 articles were selected involving 56 authors. Notably, some authors made significant contributions. Specifically, authors Parkinson, S., Pastor-Montaño, M.G., Smith, S., and Souto, M.J. collaborated on two articles each, while others contributed to only one. Excluding the five authors who wrote two articles each, the final count of authors involved in the study is 51.

The study findings reveal that authors primarily hail from Spain, comprising the largest cohort at 33.3% (*n* = 17) of the total. British and Australian authors each represent 19.6% (*n* = 10). Conversely, Canadians and North Americans contribute 9.8% (*n* = 5) each, while South Africans make up 5.9% (*n* = 3). Icelanders and Israelis are involved at 3.9% (*n* = 2) each, with authors from Hong Kong contributing 2% (*n* = 1). Statistical analysis indicates a significant difference between Spanish authors and those from Iceland, Israel, South Africa, the United States, and Canada (*p* < 0.005), suggesting a noteworthy association between author nationality and their involvement in articles on occupational therapy in UHP in these specific countries. However, no statistically significant differences (*p* < 0.005) were found, suggesting insufficient evidence to establish significant associations between author nationality and their participation in articles in the other analyzed countries.

In terms of authorship collaboration, 37.5% (*n* = 9) of articles are authored by a single individual, while 62.5% (*n* = 15) involve at least two authors. The article with the highest number of authors, five, corresponds to two articles (Enguita-Flórez et al., 2021; Evatt et al., 2016).

Regarding authorship averages, with a total of n = 24 selected articles and *n* = 51 authors, the average number of authors per article is calculated at 2.3. The historical evolution of this average indicates a 270% increase (2.5 authors) from an average of *n* = 1 author in 1986 to an average of *n* = 3.7 authors in 2021. The years with the highest average authorship are 2008 and 2011 with *n* = 4 authors, compared to 1986, 1990, 1996, 1999, 2002, and 2006, which had the smallest average of *n* = 1.

### Data Extraction on Methodological Variables in Occupational Therapy Research Articles in UHP

Regarding the study type, empirical studies demonstrate statistical significance (*p* = 0.001) compared to non-empirical ones (Table 3).

**Table 3:** Number of articles classified according to the methodology used

	nº	%	1986–95 n = 2	1996–2005 n = 3	2006–15 n = 9	2016–23 n = 10
Empirical Studies						
Qualitative						
Narrative	1	4,2				1(4.2)
Phenomenological	8	33,3		1(4.2)	4(17.6)	3(12.5)
Action Research	2	8,3		1(4.2)		1(4.2)
Quantitative						
Experimental Pre- Experimental	2	33,33	1(4.2)			1(4.2)
Non-Experimental Cross-Sectional Descriptive	4	66.67	1(4.2)	1(4.2)		2(8.4)
Mixed Methods						
Sequential Explanatory Design	1	4,2				1(4.2)
Non-Conclusive	1	4,2				1(4.2)
Non-Empirical Studies						
	5	20,8			4(17.6)	1(4.2)

Regarding the study type, empirical studies demonstrate statistical significance (*p* = 0.001) compared to non-empirical ones (Table 3).

In terms of empirical studies, qualitative articles (45.8%, *n* = 11) outnumber quantitative and mixed-method articles in occupational therapy. However, there's no statistical significance (*p* = 0.086) between qualitative and quantitative studies. Conversely, there's significance (*p* = 0.001) when comparing qualitative to mixed-method studies. Further analysis by decades shows a rise in qualitative methodologies post-1995, though not significant (*p* = 0.011). Phenomenological approaches dominate (41.7%, *n* = 10), showing significance (*p* = 0.001) compared to narrative studies, not the same with action research (*p* = 0.010). Quantitative studies (25%, *n *= 6) span all decades except 2006–2015. Mixed-methods sporadically emerge in 2016–2023 (4.2%, *n* = 1).

Non-empirical studies account for 20.8% (*n* = 5) from 2006 onward, with 2006–2015 having 17.6% (*n* = 4). However, no statistical significance (*p* = 0.058) suggests the difference in non-empirical studies between decades lacks strength for significant association.

In terms of the study population, 87.5% (*n* = 21) of the articles focused on users of occupational therapy services in these facilities, while 8.3% (*n* = 2) targeted professionals, and 4.2% (*n* = 1) aimed at both professionals and users.

Considering that two out of the 24 selected articles did not provide data on the study population and that five were theoretical articles, the remaining 17 articles involved a total of 370 individuals studied. Regarding gender representation in the research, only 10 articles collected this information, totaling *n* = 209 individuals identified by sex.

Statistical analysis indicates significantly higher representation of female authors in published articles on occupational therapy in UHP, constituting 66% (*n* = 138) of the total compared to male participation. The statistical significance value (*p* = 0.001) suggests this difference in the proportion of female and male authors is not random, indicating a noteworthy association between sex and participation in research in this specific area.

### Data Extraction on Research Outcome Variables in Occupational Therapy Articles in UHP

Regarding the diagnosis of individuals in the articles, they were classified according to DSM-V criteria (APA, 2013). The most prevalent diagnoses were schizophrenia and other psychotic disorders at 22.2% (*n* = 8), followed by depressive disorders at 16.7% (*n* = 6), substance-related and addictive disorders at 11.1% (*n* = 4), personality disorders and bipolar disorder at 8.3% (*n* = 3) each, and trauma and stressor-related disorders, and obsessive–compulsive disorder at 2.3% (*n* = 1) each. None of the analyzed articles mentioned an occupational diagnosis.

Regarding the devices, all 24 published articles used the UHP. However, a slight difference was detected among the articles: *n* = 23 (95.83%) belong to UHP, and *n* = 1 (4.17%) to UHP for adolescents.

Concerning occupational therapy interventions in the *n* = 24 selected articles, *n* = 8 did not specify any interventions carried out. Among the rest, a total of *n* = 21 interventions were compiled (Table 4), with some articles including multiple types of interventions, totaling *n* = 33. Group interventions significantly predominated (*p* = 0.001) at 75.8% (n = 25) compared to individual interventions (21.2%; *n* = 7) and family interventions (3%; *n* = 1). The interventions were categorized following those described by the "Occupational Therapy Practice Framework: Domain and Process (4th ed)" (AOTA, 2020) (Table 4), and significantly (*p* = 0.002), interventions involving occupation and activities prevailed over those supporting occupation (18.2%; *n* = 6), not the case for those related to education and training (27.3%; *n* = 9). Regarding group activities, those linked to occupation and activities were the most numerous (42.4%; *n* = 14) compared to those related to education and training (15.2%; *n* = 5). Conversely, in individual interventions, the most numerous were those related to education and training (12.1%; *n* = 4) compared to occupation and activities (6%; *n* = 2).

**Table 4:** Grouped interventions taking into account the Occupational Therapy Practice Framework: Domain and Process (4th ed)

	Occupation and activities *n* (%)	Interventions to support occupation *n* (%)	Education and training *n* (%)
Grupal			
Recovery	2(6.1)		
Actividade	4(12.1)		
Photovoice	1(3)		
Psicoeducación			1(3)
Directive group			1(3)
Physical activity	3(9.1)		
Recreational activities	2(6.1)		
Support groups			1(3)
Social activities	1(3)		
Occupational connections	1(3)		
Health education	1(3)	1(3)	2(6.1)
Soccer	1(3)		
Others		3(9.1)	
Individual			
Kitchen	1(3)		
Personal care activities	1(3)		
Reflection on occupation		1(3)	
Recreational activities			1(3)
Counseling			1(3)
Health education			1(3)
Others		1(3)	
Familiar			
Psychoeducational support			1(3)

To evaluate content relevance, we conducted a comprehensive analysis of keywords present in the *n* = 24 articles under study. The most recurrent keyword is "terapia ocupacional" (occupational therapy), occurring in 10.9% (*n* = 10) of the articles. Following closely is "salud mental" (mental health), emerging in 7.6% (*n* = 7). Additionally, "investigación cualitativa" (qualitative research) appears in 4.4% (*n* = 4) of articles. Other keywords such as "unidad psiquiátrica aguda" (acute psychiatric unit), "pacientes hospitalizados" (hospitalized patients), and "psiquiatría" (psychiatry) each occur in 3.3% (*n* = 3) of articles. Expressions like "unidad de estancia corta" (short-stay unit), "hospitalización" (hospitalization), "enfermedad mental" (mental illness), "ocupaciones" (occupations), "recuperación" (recovery), "modelo de recuperación" (recovery model), and "espiritualidad" (spirituality) gather a 2.2% (*n* = 2) occurrence. Other identified keywords do not surpass the 1% threshold, providing additional and specific nuances to the investigated set.

After reviewing the 24 intervention studies on occupational therapy in UHP and categorizing the data through abductive reasoning, four categories emerged (Table 5). Experiences of Users and Professionals in UHP (37.5%; *n* = 9), Occupational Therapy Interventions to Improve Mental Health (33.3%; *n* = 8), and Importance of Occupational Therapy in UHP (20.8%; *n* = 5) are the most predominant categories in the study of occupational therapy in UHP. Most studies were at level four of evidence (JBI, 2014) for effectiveness (75%; *n* = 18) (Observational-Descriptive Studies), except for Lipskaya-Velikovsky et al. (2016), which was an experimental design (Randomized Controlled Trial), and Lloyd et al. (2017), which corresponds to a Systematic Review of Expert Opinion. At level three of significance (JBI, 2014), there were unique qualitative studies (Table 4), and only two in Oladottir and Palmadottir's (2017) study, which corresponded to a sequential explanatory design.

- Importance of Occupational Therapy in UHP is evident from various studies. Bailliard et al. (2021) show how occupational reflection positively influences the recovery of individuals with bipolar, depressive, and addiction disorders. Synovec (2015) highlights how implementing the "recovery" model within occupational therapy enhances the quality of psychiatric care in UHP. Best (1996) demonstrates the effectiveness of occupational therapy in UHP by promoting routines and activities with profound meaning. Users themselves recognize the clear value and benefits of occupational therapy in UHP (Birken & Bryant, 2019). Additionally, group occupational therapy activities are acknowledged as useful and satisfying tools for individuals dealing with depressive disorder (Ramano et al., 2021).
- The spiritual dimension in UHP becomes significantly relevant for users, exerting a influence on their recovery process (Smith & Suto, 2014). Professionals recognizing and understanding spirituality emerge as crucial aspects in providing holistic care in UHP environments (Suto & Smith, 2014). Moreover, incorporating spirituality training could enhance the quality of care provided in these settings, with recommendations for further exploration through methodologies like broader surveys (Suto & Smith, 2014).
- Findings on User and Professional Experiences in UHP reveal the immense value of considering an occupational perspective in the recovery process, fostering user autonomy (Kennedy & Fortune, 2014). Psychoeducational model-based approaches, like creating specific groups, show promise in UHP contexts (Eaton, 2002). Occupational therapy activities consistently yield high satisfaction for both users and professionals in these environments (Enguita-Flórez et al., 2021). Identifying and analyzing user strengths and weaknesses is crucial for tailoring interventions to individual needs (Evatt et al., 2016). In line with this, Occupational therapists not only intervene at an individual level but also significantly influence mental health systems overall, emphasizing their broad impact (Castillo, 2006). Also, entering a UHP involves personal and social deconstruction, highlighting the importance of meticulous acclimatization to promote active user participation (Pastor-Montaño et al., 2019). In this context, the role of occupational therapists in UHPs encompasses their functions and fundamental aspects of occupational therapy (Sesé et al., 2011). Occupational therapists' unique perspectives enrich the understanding of challenges and opportunities in providing therapy in UHPs (Souto-Gómez & Talavera-Valverde, 2015a, b). Detailed specifications of occupational therapy services in UHP offer essential context for clinical practices and approaches (Méndez-Mena, 2006).
- Results on Occupational Therapy Interventions to Enhance Mental Health highlight the effectiveness of interdisciplinary group programs in UHP, as demonstrated by Kaplan (1986). Assessment tools like the Activity Pattern Indicator and MOHOST prove invaluable for identifying patterns and improving users' quality of life (Larson, 1990; Parkinson et al., 2008). Additionally,Occupational therapy not only enhances quality of life but also improves emotional well-being and promotes participation in meaningful activities (Parkinson, 1999). Integration into therapeutic groups, such as the football group studied by Pascual (2020), enriches users' recovery process. Even, The "Occupational Connections" intervention is highly effective in improving mental health and quality of life (Lipskaya-Velikovsky et al., 2016). In fact, Occupational therapy emphasizes holistic aspects for optimal care (Lloyd et al., 2017). So, Client-centered practice and establishing a strong therapeutic relationship are essential for guiding intervention decisions (Óladóttir & Pálmadóttir, 2017). Thus, these elements converge to promote a personalized and effective approach to therapeutic care in UHP settings.

**Table 5:** Characteristics of the articles included in the scoping review

AU/Y/TA/C	MD/PO/PC/DGTO	TI/R	C/FL	JBIe	
				LEF	LMF
Experiences of Users and Professionals in UHP					
-Eaton (2002) -Empirical -UK	-Qualitative Action Research. -An occupational therapist, a psychologist, and a nurse established a specific group of women based on a psychoeducational model. The role of the occupational therapist is analyzed -12 users (12 women) -N/A	-Group-Based: Education and Training -There is a need for greater involvement of occupational therapists in the psychological education of users in acute care units	-There is a role for occupational therapists in psychoeducation in acute mental health units; providing information and supporting active participation -N/A	4.d	3
-Castillo (2006) -Theoretical -Spain	-N/A -Addressing the Importance of Occupational Therapy in a Short-Term Hospitalization Unit, its Integration in a Holistic Therapeutic Approach, and its Relationship with Other Treatments -Users (number not specified) (gender not specified) -N/A	-Group-Based: Occupations and Activities -N/A	-Occupational therapy is a fundamental intervention in the recovery of users admitted to a short-term hospitalization unit -N/A	N/A	5
-Méndez-Mena (2006) -Theoretical -Spain	-N/A -Describing a Short-Term Hospitalization Unit for Adolescents -Users (number not specified) (gender not specified) -N/A	-Group-Based: Occupations and Activities -They describe the characteristics of the short-term hospitalization unit and its management. They highlight the advantages of short-term hospitalization	-The short-term hospitalization unit is a viable option for the treatment of adolescents with mental health issues, always considering their needs and involving the family -N/A	N/A	5
-Sesé et al. (2011) -Theoretical -Spain	-N/A -Detailing the Role and Objectives of Occupational Therapist Intervention within the Acute Psychiatry Unit. Additionally, Presenting the Therapeutic Activities Program -Users (number not specified) (gender not specified) -N/A	-Group-Based: Education and Training, Occupations and Activities, Interventions to Support Occupations, Others. Individual: Occupations and Activities -The occupational therapy program was effective in improving the functional status of the users	-Occupational therapy is a valuable intervention for improving the functional status and quality of life of users with psychiatric disorders admitted to acute care units -N/A	N/A	5
-Souto-Gómez & Talavera-Valverde (2015b) -Theoretical -Spain	-N/A -Narrating the Experience of an Occupational Therapist in a Short-Term Hospitalization Unit to Address the Lack of Homogeneity in Interventions -1 Professional (1 woman) -N/A	-N/A -N/A	-N/A -N/A	N/A	5
-Kennedy & Fortune (2014) -Empirical -Australia	-Qualitative Phenomenological -Identifying Factors Influencing Occupational Engagement of Female Users in a Mental Health Unit in Australia -6 Users (6 Women) -Schizophrenia and other psychotic disorders, Personality Disorder, and others (symptoms)	-N/A -Women admitted to an acute psychiatric unit experience a loss of autonomy and control over their daily lives, which can have a negative impact on their mental health	-Taking into account the occupational perspective helps individuals regain control of their lives and promote mental health - Expanding the research scope to include the experience of improving psychiatric spaces	4.b	3
-Evatt et al. (2016) -Empirical -Australia	-Quantitative Non-Experimental, Cross-Sectional, Descriptive -Analyzing collected data to understand consumer functioning in HDU (High Dependency Units) and PICU (Psychiatric Intensive Care Units) -70 users (gender not specified) -Substance-related disorders and addiction	-N/A -There may be some conceptual overlap between adjacent categories, and the pattern of recovery of functional capacities, as described in the Hyperacute Screening Tool (HST), may not be applicable to a minority of individuals	-Strengths and weaknesses of users help occupational therapists develop optimal interventions -Explore the functionality of users in other HDU and PICU and evaluate the measurement properties of the HST	4.b	N/A
- Pastor-Montaño et al. (2019) -Empirical -Spain	-Qualitative Phenomenological -Understanding and delving into the experience of admission to a UHP from the perspective of the user -12 users (4 women, 8 men) -N/A	-N/A -It is important to address the occupational needs of users during their stay and consider their opinions and preferences in treatment planning	-Admission to a UHB (Unmanned Hydrographic Vehicle) causes personal and social disorientation. Facilitating this adjustment during admission and subsequent adaptation to discharge is crucial -N/A	4.b	3
-Enguita-Flórez et al. (2021) -Empirical -Spain	-Quantitative Non-Experimental, Cross-Sectional, Descriptive -Investigating satisfaction in a Short-Term Stay Unit (STSU) regarding occupational therapy activities -64 users and professionals (39 women, 25 men) -Schizophrenia and other psychotic disorders, Personality Disorder, Substance-related disorders, and addiction	-Group: Occupations and Activities; Individual: Education and Training -The study results indicate a high level of user satisfaction with occupational therapy	-Occupational therapy is a valuable intervention for users admitted to an acute psychiatric hospitalization unit -Future study to reflect on progress in the unit	4.b	N/A
Occupational Therapy Interventions to Improve Mental Health					
-Kaplan (1986) -Empirical -USA	-Quantitative Experimental, Pre-Experimental -Describing a three-tier interdisciplinary group program to treat users -12 users (gender not specified) -Depressive disorder, Other (symptoms)	-Group: Others -Significant improvement in users treated with the Directive Group compared to conventional occupational therapy	-Directive Group is an effective and efficient occupational therapy intervention for users -A limitation of the directive group is that no follow-up was conducted	3.e	N/A
-Larson (1990) -Empirical -USA	-Quantitative Non-Experimental, Cross-Sectional, Descriptive -Using the Activity Pattern Indicator (API) (Diller, Fordyce, Jacobs, and Brown, 1978) and the Schedule of Recent Experience (SRE) (Holmes, 1981) to determine activity patterns and life changes in users with depression -15 users (7 women, 8 men) -Depressive disorder	-Group: Occupations and Activities -Individuals with depression tend to have limited activity patterns and experience significant changes in their lives	-Occupational therapy plays a significant role in assisting individuals with depression to improve their quality of life -Further research with a larger population and analysis of elements of the Activity Pattern Indicator (API) is necessary	4.b	N/A
-Parkinson (1999) -Empirical -UK	-Quantitative Non-Experimental, Cross-Sectional, Descriptive Audit of the occupational therapy service in an Acute Mental Health Unit at Chesterfield and North Derbyshire Royal Hospital NHS Trust -N/A -N/A	-Group: Occupations and Activities -The group program was well-received by patients and helped reduce anxiety while improving mood and participation in activities	-Group programs for users admitted to a mental health unit are useful for improving their emotional well-being and participation in meaningful activities -N/A	4.b	N/A
-Parkinson et al. (2008) -Empirical -UK	-Quantitative Case Study -Case study and discussions with occupational therapists to explore how assessment influences practice and how MOHOST-based observation forms contributed to the process -1 user (1 woman).** -N/A	-N/A -MOHOST assessment it is a useful tool for assessing the occupational performance of users with psychiatric disorders in an acute hospital setting	-MOHOST assessment it is a useful tool for planning and implementing occupational interventions -In the future, it will be considered how to leverage the richness of the data to investigate the effectiveness of occupational therapy services	4.d	N/A
-Lipskaya-Velikovsky, et al. (2016) -Empirical -Israel	-Quantitative Experimental, Pre-Experimental -Describing a structured intervention called Occupational Connections (CO/OC) -10 users (gender not specified) -Spectrum of schizophrenia and other psychotic disorders	-Group: Others -Significant improvement in patients' perception of their quality of life and mood after the intervention	-"Occupational Connections" is a useful intervention for improving mental health and quality of life -More participants and a control group will be included. The effect of other medications and treatments will also be studied	1.c	N/A
-Lloyd et al. (2017) -Theoretical -Australia	-N/A -Providing a reflection on how occupational therapists can influence mental health systems to collaborate with other professionals -Users (number and gender not specified) -N/A	-Group: Others -The results indicate that occupational therapy can play a significant role in promoting a stronger and more effective peer workforce	-Occupational therapy contributes to recovery-oriented care, and intervention programs should be developed to support the peer workforce -N/A	5.a	4
-Óladóttir & Pálmadóttir (2017) -Empirical - Iceland	-Mixed-Methods: Explanatory Sequential Design -Examining the perceptions of mental health users regarding the care they received in a hospital setting -30 users (19 women, 11 men) -Bipolar disorder, Depressive disorder, Personality disorder, Schizophrenia and other psychotic disorders, Other (symptoms)	-N/A -Client-centered practice is perceived as important by users. Additionally, users' perceptions vary based on their level of involvement in decision-making regarding their treatment	-Client-centered practice is a useful approach, and steps should be taken to improve user decision-making -Further research is needed on the perspectives of users and professionals, taking into account different institutional forces	4.b	2
-Pascual (2020) -Empirical -Spain	-Qualitative Action Research -Analyzing the therapeutic football group as an empowerment tool within the sports program in a hospitalization unit -N/A -N/A	-Group: Occupations and Activities. -Participation in the therapeutic football group aids in the recovery and empowerment of users	-Engaging in sports is a useful tool in occupational therapy for users admitted to acute psychiatric units. -N/A	4.b	3
Importance of Occupational Therapy in UHP					
-Best (1996) -Empirical -UK	-Qualitative Phenomenological -Providing an example of a successful provision of occupational therapy services in a Psychiatric Intensive Care Unit (PICU) and describing the challenges for the therapist -1 user (1 man) -Schizophrenia and other psychotic disorders	-Individual: occupations and activities - A relationship of mutual trust was established and the user’s functional, cognitive, and mental state were evaluated	-Occupational therapy plays an important role in the care of users admitted to UCIP. The author also highlights the need for collaboration and coordination among mental health professionals -N/A	4.b	3
-Synovec (2015) -Empirical -USA	-Qualitative Phenomenological -Identifying the effectiveness of occupational therapy using the principles of the recovery model from the perspective of users within a psychiatric inpatient unit -53 users (gender not specified) -Depressive disorder, Bipolar disorder, Schizophrenia and other psychotic disorders, Trauma-related and stressor-related disorders, Obsessive–compulsive disorder, Substance-related disorders and addiction, Dual disorders	-Group: Others -The implementation of the principles of the recovery model in occupational therapy can be beneficial for hospitalized users with psychiatric disorders	-Applying the principles of the recovery model can improve the quality of psychiatric care -Future research should use a mixed methodological approach, and follow-up after admission may also be beneficial	4.b	3
-Birken & Bryant (2019) -Empirical -UK	-Qualitative Narrative -Investigating how an occupational therapy department with specific facilities is experienced by service users in an acute mental health unit in the London Borough -17 users (gender not specified) -N/A	-Group: Occupations and activities -Users perceive occupational therapy to be valuable and beneficial to their recovery" in English	-The participation of users in the therapeutic process is essential to meet their needs and improve their hospital experience -N/A	4.b	3
-Bailliard et al. (2021) -Empirical -USA	-Qualitative Phenomenological -Exploring whether participating in an occupational reflection intervention in an inpatient psychiatric unit can support the recovery of adults living with severe mental illness -10 users (4 women, 6 men) -Bipolar disorder, Depressive disorder, Substance-related disorders, and addiction	-Individual: Others -Structured reflection improved recovery by understanding how occupations affect mental health	- Occupational reflection can favor the recovery of adults living with serious mental disorders -Future research should have a larger sample size and use standardized assessments	4.b	3
-Ramano et al. (2021) -Empirical -South Africa	-Qualitative Phenomenological -Describing the perceptions and experiences of hospitalized adult psychiatric users with major depressive disorder towards activity-based groups in occupational therapy -50 users (43 women, 7 men) -Depressive disorder	-Group: Occupations and Activities -As activities of occupational therapy based on groups were perceived as useful, relevant, and satisfactory by the users	-Group-based occupational therapy is a useful tool for treating users with major depressive disorder -The intervention should be investigated in a public health context and evaluate the influence of other treatments, carry out a follow-up	4.b	3
Spirituality and Meaning in UHP					
-Smith & Suto (2014) -Empirical -Canadá	-Qualitative Phenomenological -Part 1 of an investigation exploring the experience of spiritual conversations for mental health users and professionals. 7 users (2 women, 5 men) N/A	-N/A -Spirituality is a frequent topic among users, and it can play an important role in their recovery	-Mental health professionals should take into account spirituality and encourage its exploration and discussion in clinical contexts -N/A	4.b	3
-Suto & Smith, (2014) -Empirical -CanadA	-Qualitative Phenomenological -Part 2 of an investigation exploring the experience of spiritual conversations for mental health professionals -Professionals (number and gender not specified) -N/A	-N/A -Spirituality is an important topic for many patients in acute psychiatric units, and mental health professionals are open to discussing this topic with the people they treat	-The incorporation of spirituality in mental health care is beneficial for patients and the training of professionals, improving the quality of mental health care -Surveys will be included in future research	4.b	3

*AU/A:* author/year; *TA:* Type Article; *C:* Country; *MD/PO/PC/DGTO:* Methods and desing/Research question or objectives/ type of population and quantity/ diagnoses used; *TI/R:* Types of interventions used/ results; *C/FL:* Conclusion/Future lines of research; *JBIe:* JBI level of evidence; *LEF:* Level of evidence for effectiveness; *LMF:* level of evidence for meaningfulness

## Discussion

The research results have successfully met the overarching goal of identifying scientific publications on occupational therapy in UHP. The cohesive integration of these findings in the discussion section offers clear and well-supported responses to the specific objectives outlined.

### Historical Analysis of Occupational Therapy Research in Mental Health

The evolution of research in occupational therapy and mental health is notable, showcasing achievements and laying a robust foundation for future investigations. Understanding historical trends, diverse contributions, and research drivers is crucial for advancing in this critical field for mental health. A global, inclusive, and multidisciplinary approach is essential for sustainable development and ongoing effectiveness of occupational therapy in mental health care settings, especially in UHP.

#### Historical Trends and Temporal Development

The historical progression of scientific literature on occupational therapy in UHP offers valuable insights into the field's development, reflecting trends observed in other health sciences (O'Brien, 2001). Starting modestly in 1943 and peaking with 234 publications from 2013 to 2023, there's notable advancement (p = 0.001). This growth is attributed in part to evolving mental health understandings (Johnson, 2021) and wider recognition of occupational therapy's efficacy in these contexts (March, 2017; Whitley et al., 2011). This upward trend is also influenced by progress in health sciences disciplines, aiming to forge new research paths to deliver quality, efficient, multidisciplinary, holistic, and humane care (March, 2017).

#### Temporal Analysis and Contributing Factors

Decade-wise analysis reveals intriguing patterns, notably a substantial increase in the last two decades. There's a statistically significant rise (p = 0.001) in published articles during 2003–2012 and 2013–2023 compared to preceding decades, indicating an intensified research focus on occupational therapy in UHP.

The findings yield valuable insights into the evolution of occupational therapy research and its connection with UHP over time. This trend is influenced by several factors: (a) Changes in clinical care and mental health policies (Johnson, 2021), alongside improved understanding of mental health issues and increased acceptance of occupational therapy methods (Wainberg et al., 2017); (b) Embracing the digital age (Vessuri, 2014) and globalization, which offer new tools to expedite scientific literature production; (c) The rise in publications by organizations like Organización Mundial de la Salud (OMS) (1996) and WFOT (2019), advocating mental health care principles and less restrictive approaches, thus promoting occupational therapy and research (Mapanga et al., 2019); (d) Evolving clinical care and technological advancements stimulating research (Lau et al., 2020; Lau et al., 2021); (e) Lastly, the notable increase in occupational therapists with master's and doctoral degrees, starting in the US in 1997 with the master's degree becoming the basic level of education. This shift likely influenced subsequent research growth. A similar pattern emerged in the EU post-2005 with the introduction of university master's programs and doctoral degrees, facilitated by the European Higher Education Area and Bologna Process reforms (European Ministers of Education, 1999; Polonio, n.d.). These reforms transformed former diploma titles into bachelor's degrees, expanded access to postgraduate education, including master's programs and doctoral studies.

#### Document Typology and Diversity of Contributions

A high proportion of documents published in indexed journals (98.6%) indicates rigorous and high-quality research in this field, emphasizing the robustness of the analyzed scientific literature, primarily sourced from esteemed academic outlets (Sobrido-Prieto et al., 2019). Although most documents adhere to journal formats, the inclusion of other formats like book chapters and doctoral theses indicates diverse approaches, reflecting the richness of contributions shaping the advancement of knowledge in occupational therapy at this intervention level (Piedra & Martínez-Rodríguez, 2007).

#### Linguistic Dimension and Global Dissemination

The predominance of English as the primary publication language (87.2%), supported by statistical significance (p = 0.004), highlights the Anglophone community's sway in research. This ties to factors like journal internationalization, audience targeting, and occupational therapy research accessibility (Sobrido-Prieto et al., 2021, 2023). This underscores the significant impact of the Anglophone scientific community on UHP occupational therapy research (Brown et al., 2005). Franco-López et al. (2016) suggest that language choice significantly influences citation rates and article impact factors, given English's dominance in influential journals, promoting researchers' professional growth through publication in this language.

While English as the primary scientific communication medium aids global dissemination, concerns arise regarding global representation and the need for strategies promoting inclusion of diverse languages and cultural perspectives (Navarro, 2001). English's linguistic hegemony may impede effective communication and result in knowledge loss (Lopes et al., 2018). We acknowledge the importance of valuing studies in different languages to promote a global understanding of occupational therapy in UHP. Although the presence of articles in languages other than English (such as Spanish) is less frequent in our study, we believe we have reflected cultural and linguistic diversity in research at this level of intervention (Castillo, 2006; Méndez-Mena, 2006; Sesé et al., 2011; Souto-Gómez & Talavera-Valverde, 2015b; Pascual, 2020; Pastor-Montaño et al., 2019; Enguita-Flórez et al., 2021).

### Synthesis of Evidence on Occupational Therapy Literature in UHP

An in-depth examination of articles on occupational therapy in UHP offers a comprehensive and contextualized perspective on research in this domain. The analysis underscores global interest continuity, geographical diversity, language preferences, and evolving collaboration and authorship patterns, indicating the expanding complexity and scope of research in occupational therapy within this clinical context. These insights provide a solid groundwork for future research and guide the trajectory of occupational therapy in UHP.

#### Increase in Scientific Production on UHP and Occupational Therapy

Research on occupational therapy in psychiatric UHP displays an upward trajectory, marking a significant milestone in this field's advancement (p = 0.001). This growth reflects an increasing acknowledgment of occupational therapy's efficacy in acute mental health interventions (Christie et al., 2021; Scheewe et al., 2013). Three key factors contribute to this rise: (a) growing recognition of occupational therapy's effectiveness in acute settings (Christie et al., 2021; Scheewe et al., 2013), (b) integration of occupational therapy programs into mental health university curricula (Arblaster et al., 2015; Scanlan et al., 2017), and (c) WFOT's (2019) advocacy for integrating occupational therapy into mental health care and promotion, potentially fostering researcher and professional interest in this domain.

#### Temporal Distribution of Publications

Analysis of research on occupational therapy in UHP across time demonstrates consistent presence without a clear trend towards increased or decreased interest (Gallagher et al., 2023). This suggests occupational therapy's enduring relevance in mental health settings, likely owing to its recognized positive impact on recovery (Gallagher et al., 2023; Zango-Martín et al., 2022). Amid 446 identified publications on occupational therapy in UHP during the study period, 24 articles met analysis criteria. Variable production over time indicates sustained interest in occupational therapy in UHP without a distinct temporal trend.

The lack of statistical significance in comparing publication decades reflects occupational therapy's adaptable nature, accommodating evolving needs and clinical approaches over time (Gallagher et al., 2023; Moruno-Miralles & Talavera-Valverde, 2011). This underscores occupational therapy's dynamism, capable of evolving to meet evolving demands in mental health care (Hyett et al., 2019).

#### Geographical Context of Publications

Spain leads in geographical distribution of studies with 29.2%, followed by the United Kingdom (20.8%), the U.S. (16.7%), Australia (12.5%), and Canada (8.3%). Iceland, Israel, and South Africa, each contribute 4.1%. Though not statistically significant, these variances emphasize the importance of cultural and contextual considerations when applying findings across diverse clinical settings (Hyett et al., 2019). Geographical diversity enriches understanding of successful interventions and best practices in occupational therapy within UHP (Gallagher et al., 2023; Hyett et al., 2019).

#### Nationality of Authors

Disparity in authors' nationalities underscores cultural influence on UHP occupational therapy research. Statistical significance between Spanish authors and those from specific countries emphasizes the importance of cultural contextualization in interpreting results. Collaboration among researchers of varied nationalities enhances research with unique perspectives and clinical experiences (Aceituno-Aceituno et al., 2015).

The prominence of Spanish authors in UHP occupational therapy publications is notable (Castillo, 2006; Méndez-Mena, 2006; Sesé et al., 2011; Souto-Gómez & Talavera-Valverde, 2015b; Pascual, 2020; Pastor-Montaño et al., 2019; Enguita-Flórez et al., 2021). This likely reflects the leadership and interest of the Spanish academic community in this area, raising questions about cultural and contextual influences on research. Factors explaining this include: (a) variations in UHP characteristics and approaches across countries possibly excluding some articles from this review  (Moruno-Miralles & Talavera-Valverde, 2011); (b) Spain's mental health system and occupational therapy practices shaping research direction and focus, fostering unique leadership and perspectives (Ministerio de Sanidad, 2021; Zapata-Moya & Navarro-Yáñez, 2021); (c) lower author representation from other countries due to differing contexts and research concentration. This highlights the importance of cultural diversity (James & Prilleltensky, 2002) when generalizing international study results and the need to explore regional variations in occupational therapy program implementation.

#### Journals and Impact Factors

The 24 papers span 13 peer-reviewed journals. While 53.8% of these journals appear in both the Journal Citation Report (JCR) and Scimago Journal Ranking (SJR), no statistical significance (p > 0.005) is found in comparison to those not listed in these indices. However, it's noteworthy that Q3 quartile journals dominate in both JCR and SJR, indicating consistent publication quality. Despite no significant association (p > 0.005) between inclusion in JCR/SJR and journal quality, this difference may be due to chance rather than a true correlation between impact factors and inclusion (Brown & Gutman, 2019; Brown et al., 2018). This suggests that a journal's presence in these indices doesn't significantly affect analyzed characteristics or attributes (Johnson & Leising, 1986; Sobrido-Prieto et al., 2021), though other unanalyzed factors may contribute. Nevertheless, publication in peer-reviewed journals implies a standard of research quality and rigor (Sobrido-Prieto et al., 2021).

The diversity of journals and their inclusion in various impact indices underscores the necessity for comprehensive evaluation of publication quality, factoring in impact, clinical relevance, and innovation (Sobrido-Prieto et al., 2021). The preference for established journals like the British Journal of Occupational Therapy (25%) and the American Journal of Occupational Therapy (16%) among authors reflects the occupational therapy community's favor for recognized platforms (Sobrido-Prieto et al., 2021, 2023).

#### Authorship and Collaboration Characteristics (Multidisciplinary Collaboration and its Impact on Research Quality)

The 24 articles had 56 authors, some contributing to multiple articles. Spanish authors represented 33.3%, followed by British and Australian authors (19.6% each). A statistically significant correlation between authors' nationality and their participation in specific countries' articles (p < 0.005) highlights cultural and contextual influence (James & Prilleltensky, 2002) on UHP occupational therapy research.

Detailed analysis shows significant collaboration among authors in UHP occupational therapy research, with 62.5% of articles authored by at least two individuals, emphasizing the need for complementary skills and multidisciplinary perspectives (Sobrido-Prieto et al., 2023). The increase in average authors per article over time, from 2.5 in 1986 to 3.7 in 2021, suggests a trend towards collaborative authorship, in line with previous research (Sobrido-Prieto et al., 2023).

The prevalence of collaborative authorship raises questions about research dynamics and quality. The high rate of collaborations (62.5%; n = 15) underscores the preference for collaborative research in UHP occupational therapy (Comité Gestor del Consejo de Colegios de Terapia Ocupacional. Asociaciones Profesionales de Terapia Ocupacional, 2013). This trend, supported by studies like Sobrido-Prieto et al. (2023) and Wuchty et al. (2007), mirrors the broader shift towards larger research teams across disciplines. While multidisciplinary collaboration can foster innovation, it's crucial to scrutinize the assumption that more authors equate to higher scientific quality (Valderas et al., 2007). Establishing objective standards for evaluating collaborative research quality in occupational therapy is essential (Valderas et al., 2007) to ensure credible and actionable findings for clinical practice and mental health policy formulation.

The increasing trend towards collaboration over time suggests that the complex challenges of mental health care demand integrated approaches and specialized teams (Brown et al., 2018). This trend may be attributed to the growing need for diverse expertise in addressing mental health complexities and occupational interventions (Brown et al., 2018).

### Detailed Volume of Scientific Literature on Occupational Therapy in UHP

#### Types of Studies

The analysis of occupational therapy articles in UHP reveals significant findings and areas of interest, with a preference for empirical approaches, emphasizing the importance of research-based evidence (Fusar-Poli et al., 2020; Tomlin & Borgetto, 2011).

Regarding non-empirical studies, though there's an upward trend, the lack of statistical significance (p = 0.058) suggests that observed variations between decades could be due to chance. Insufficient evidence exists to assert a significant association between decades and the quantity of non-empirical studies in occupational therapy (Aylott et al., 2019).

Within empirical studies, qualitative methodologies predominate (45.8%, n = 11), with phenomenology being the most common approach (41.7%, n = 10) from 1996 onwards (Borell et al., 2012). This emphasis on phenomenology likely reflects an interest in understanding subjective dimensions of occupational therapy in short-stay hospital environments, aligning with the holistic nature of the discipline (Jack, 2006).

Over time, there's a shift towards qualitative approaches, though statistical significance isn't consistently reached (p = 0.086). However, a significant difference is found between qualitative and mixed-method studies (p = 0.001), indicating a growing interest in qualitative approaches in recent decades (Palinkas, 2014).

The prevalence of qualitative studies (45.8%, n = 11) compared to quantitative and mixed-methods studies likely results from a heightened focus on in-depth exploration of experiences and perceptions (Palinkas, 2014). Quantitative studies have remained constant, with a notable increase in the last decade (2016–2023) (Luchins, 2012). The occasional appearance of mixed-methods studies reflects methodological diversification, aiming to balance qualitative depth with quantitative objectivity (Bennett et al., 2007).

However, the choice between qualitative or quantitative methods in UHP occupational therapy research likely depends on various factors and isn't significantly associated with any specific methodology (Hitch & Lhuede, 2015).

Detailed analysis by decades reveals a transition in methodological preferences. While the first decade (1986–1995) was marked by quantitative studies predominance (Luchins, 2012), subsequent decades saw a shift towards qualitative methodologies (Borell et al., 2012), though statistical significance isn't always reached (p = 0.011). This shift may reflect the complexity of addressed phenomena, where qualitative narratives offer a holistic and contextualized understanding (Borell et al., 2012). However, the significant increase in quantitative studies in the last decade (2016–2023), along with the occasional mixed-methods studies, highlights methodological diversification and reflects an attempt to bolster evidence supporting occupational therapy interventions and practices (Bennett et al., 2007).

#### Population Studied in Occupational Therapy Research in UHP

Occupational therapy research in UHP primarily focuses on service users (87.5%, n = 21), reflecting a patient-centered orientation (Ferreira & Artmann, 2018). However, limited attention to professionals (8.3%, n = 2) and a small proportion studying both professionals and users (4.2%, n = 1) suggest an opportunity to broaden perspectives and integrate experiences of intervention implementers. While emphasizing the importance of studying user experiences for therapy quality and effectiveness (Doyle et al., 2013), scant attention to professionals indicates potential knowledge gaps and suggests future research directions. Improving user health is crucial, but humanizing and recognizing professionals can also significantly impact outcomes (March, 2017). We propose expanding the studied population to include both user experiences and the vital role of professionals in intervention effectiveness.

#### Gender Representation

Research participation in occupational therapy in UHP shows a significant female prevalence (66%, n = 138), with a statistical association (p = 0.001) between sex and field participation. This gender imbalance prompts questions about research equity and highlights the need for greater diversity to ensure result validity and relevance in mental health contexts (Morton et al., 2022).

The focus on females in most articles, unusual in research due to historical gender bias (Manterola & Otzen, 2015), is notable. In mental health, social and cultural factors influence problem development, manifesting differently based on gender roles, potentially leading to underdiagnosis in men (Montero et al., 2004). Despite these factors, considering feminist theory/perspective in future research can elucidate reasons behind this issue and achieve a more balanced view.

### Evaluating Evidence on Occupational Therapy Interventions in UHP Users' Functionality, Quality of Life, and Recovery

Thorough exploration of outcome variables in occupational therapy articles within UHP offers a detailed view, revealing complexities and essential dimensions of the discipline's application in these settings. These findings underscore areas for improvement, such as the need for detailed intervention descriptions and exploration of how occupational diagnosis influences therapeutic care.

#### Diagnoses

The prevalence of schizophrenia and other psychotic disorders (22.2%, n = 8) highlights the clinical complexity of UHP patients, indicating a necessity for tailored therapeutic approaches (Maj et al., 2021). Notably, while medical diagnostic terms are commonly used, the absence of occupational diagnoses is striking. This gap underscores a need for professional reasoning in understanding how occupation affects mental health in these contexts (Moruno-Miralles, 2002; Talavera-Valverde, 2015a, b). The absence of occupational diagnoses raises questions about how occupational therapy integrates into assessment and intervention selection compared to diagnosis-based medical interventions (Talavera-Valverde, 2015a, b). Addressing this gap is crucial for advancing understanding of occupational therapy in mental health contexts.

#### Setting

Consistent use of UHP as a study environment demonstrates research focus coherence. However, differences in data between adult and adolescent unit studies underscore the need to address occupational practice specifics in adolescent and youth populations in future research and clinical settings. Evolving mental health plans, policies (Johnson, 2021), and care for vulnerable populations (Daniels et al., 2022) can facilitate adolescent UHP development, an area requiring more attention compared to adult units.

#### Content and Keywords

Keyword analysis reveals prevalent themes in occupational therapy literature within UHP. Common terms like "occupational therapy" and "mental health" underscore their central role, highlighting the intrinsic connection between occupational therapy and mental health care (Kleinman, 1992). Additionally, terms such as "qualitative research," "acute psychiatric unit," and "recovery" emphasize the relevance of qualitative approaches and specific settings (Best, 1996; Borell et al., 2012; Parkinson, 1999; Synovec, 2015).

The diversity of less common keywords offers nuanced insights, including spirituality, indicating a broad exploration of human experience in occupational therapy within these settings. This reflects person-centered practice theories and the integration of occupational therapy models (Chen et al., 2020; Lee et al., 2012; Synovec, 2015; Wallengren et al., 2022).

## Categories Derived from Analysis of Empirical Studies

Analysis of 24 intervention studies in UHP reveals a diverse range of occupational therapy interventions, indicating a comprehensive approach to addressing individuals' needs in these settings (Castillo, 2006; Evatt et al., 2016; Lloyd et al., 2017; Ramano et al., 2021). This diversity highlights the importance and effectiveness of occupational therapy in this clinical context, contributing to holistic recovery.

The absence of intervention descriptions in six articles (Kennedy & Fortune, 2014; Óladóttir & Pálmadóttir, 2017; Pastor-Montaño et al., 2019; Smith & Suto, 2014; Souto-Gómez & Talavera-Valverde, 2015b; Suto & Smith, 2014) raises questions about investigation clarity and emphasizes the need for standardized practices in the literature to facilitate replicability and result comparison. These findings underscore the importance of ongoing research and innovative approaches in occupational therapy to optimize care in mental health settings. Continuous attention to users' unique experiences and constant intervention adaptation is essential to advance occupational therapy effectiveness in UHP (Moruno-Miralles & Talavera-Valverde, 2011).

Grouping interventions according to the "Occupational Therapy Practice Framework" (AOTA, 2020) highlights a significant volume of occupation- and activity-related interventions, emphasizing their importance in the therapeutic process. This prevalence likely stems from the foundational role of occupation and activities in occupational therapy (Moruno-Miralles & Talavera-Valverde, 2011).

The preference for group interventions in occupational therapy (p = 0.001), particularly those related to occupation and activities, suggests therapeutic value in collaborative and contextualized approaches, aligning with occupational therapy principles promoting inclusion and meaningful participation in daily life (Gallagher et al., 2015). This emphasis on group interventions may signal a focus on building inclusive environments, fostering social participation, and facilitating user connections (Díaz de Neira et al., 2021).

While group interventions prevail, the identification of successful individual interventions suggests room for personalized care in UHP. Balancing individualized approaches with interventions fostering social interaction and group cohesion is crucial to effectively address users' diverse needs (Radnitz et al., 2019).

Assessing methodological quality and study design is crucial. Classifying all studies according to the Joanna Briggs Institute's (JBI) (2014) level of evidence offers a uniform basis for assessing robustness. Most studies fall into the effectiveness category, characterized by observational-descriptive designs (Thiese, 2014), essential for exploring phenomena in natural settings. Despite limited diversity, methodological consistency enhances the internal validity of our review, providing a solid foundation for drawing conclusions about occupational therapy application in UHP.

From the reviewed literature, four emerging categories provide comprehensive insight into occupational therapy's contribution in mental health environments:

- Importance of Occupational Therapy in UHP: The results suggest that occupational therapy plays a crucial role in improving mental health in UHP. Occupational reflection (Bailliard et al., 2021) positively impacts the recovery of individuals affected by bipolar, depressive, and addiction disorders (Enguita-Flórez et al., 2021; Larson, 1990; Synovec, 2015). These disorders, characterized by marked impairment in daily functioning and quality of life, find valuable resources in occupational therapy to restore balance and stability. Implementing the "recovery" model in occupational therapy not only demonstrates its intervention effectiveness but also enhances mental health care quality in UHP. We believe that this approach demonstrates the effectiveness of occupational therapy in promoting performance patterns (routines) and meaningful activities. This approach, supported by studies like Lloyd et al. (2017) and Synovec (2015), focuses on purposeful activities, contributing to building identity and a sense of belonging (Parkinson, 1999; Pascual, 2020; Ramano et al., 2021). Actively integrating occupational approaches into intervention protocols positively impacts mental health care effectiveness (Moruno-Miralles & Talavera-Valverde, 2011). Users' positive perception, particularly in group activities, underscores the utility and satisfaction of occupational therapy in depressive disorder intervention (Larson, 1990; Ramano et al., 2021).
- Spirituality and Meaning in UHP: The findings stress the significance of considering spirituality in UHP to provide comprehensive care. Recognizing spirituality as a crucial element in the recovery process suggests that occupational therapy should address deeper dimensions of human experience beyond physical and psychological aspects (Smith & Suto, 2014). Spirituality, often overlooked in conventional mental health care, emerges as a valuable resource for more meaningful recovery (Smith & Suto, 2014; Suto & Smith, 2014). Understanding and acknowledging spirituality highlight essential elements for comprehensive care in UHP, emphasizing the importance of spirituality training for occupational therapy professionals emphasizing it as a potential strengthening component that provides a foundation for addressing the holistic needs of users (Huang et al., 2022; Smith & Suto, 2014; Suto & Smith, 2014).
- Experiences of Users and Professionals in UHP. Findings regarding the experiences of users and professionals in UHP underscore the significance of the occupational perspective in the recovery journey. Autonomy emerges as a valuable element, suggesting that occupational therapy empowers individuals by equipping them with tools and skills to make informed decisions and actively engage in their recovery (Pascual, 2020). Psychoeducational approaches, satisfaction with occupational therapy activities, and strengths and weaknesses analysis are highlighted as key aspects. Moreover, occupational therapists play an integral role not only at an individual level but also in influencing mental health systems. Creating specific groups shows promise in UHP contexts, offering practical insights into mental health issues and fostering communities of learning and support with lasting effects on recovery (Eaton, 2002).
  High user and professional satisfaction with occupational therapy activities underscore the importance of integrating these interventions into healthcare programs. Positive user perception, especially in group activities, highlights the utility and satisfaction of occupational therapy in depressive disorder intervention, fostering expression, mutual understanding, and support networks among users. This social aspect of occupational therapy could be vital for addressing loneliness and isolation, common factors in mental health problems (Pascual, 2020; Synovec, 2015).
- Occupational Therapy Interventions to Improve Mental Health. Results regarding occupational therapy interventions to enhance mental health demonstrate the effectiveness of occupational therapy in UHP environments. Participation and integration into therapeutic groups, such as the soccer group, suggest that recreational and sports activities can effectively improve mental health. This holistic approach, extending beyond traditional interventions, underscores the importance of considering a variety of meaningful activities in occupational therapy (Restall & Egan, 2021).

In this regard, client-centered practice and building strong therapeutic relationships emerge as essential guiding factors in the intervention process, emphasizing understanding individual user needs and adapting interventions accordingly (Restall & Egan, 2021). Occupational therapy, with its comprehensive approach (Restall & Egan, 2021), contributes not only to quality of life but also to well-being and participation in meaningful activities. The importance of client-centered practices (Óladóttir & Pálmadóttir, 2017) and strong therapeutic relationships highlights the need for a personalized and effective approach in UHP contexts. Additionally, recognizing occupational therapists as agents of change at both individual and mental health system levels emphasizes the breadth of their impact (Picotin et al., 2021).

### Future Research Directions

To deepen our understanding of trends over time, we suggest exploring historical trends identified in the review, particularly regarding the evolution of occupational therapy practices in UHP. This could provide insights into shifts in care and therapeutic approaches across different periods. Additionally, conducting cross-cultural comparisons would allow for exploration of variations and commonalities in the implementation and outcomes of occupational therapy in diverse cultural contexts. Similarly, investigating how contextual factors such as mental health policies and healthcare system structures influence the implementation and outcomes of occupational therapy in UHP could provide significant insights. Furthermore, we propose studies examining the economic impact of occupational therapy interventions, offering valuable information for informing decision-making in mental health policies.

### Limitations

The predominance of English-language publications may introduce a language bias, potentially limiting the representation of research in other languages. Furthermore, focusing solely on indexed journals could exclude relevant studies not published in these sources, potentially introducing publication bias. Acknowledging publication biases, there is a possibility that studies with negative or nonsignificant results were not published, which could skew the review towards more positive findings. Similarly, while efforts were made to conduct a comprehensive search across various databases, restricting inclusion to literature in English, Spanish, and Portuguese might have led to the omission of relevant research in other languages, potentially impacting the global representativeness of the review.

## Conclusions

This study underscores the growth, methodological shifts, and specific focus on experiences and effectiveness in occupational therapy within UHP. The scoping review demonstrates a notable increase in occupational therapy research in UHP, particularly emphasizing qualitative studies, highlighting the essential role of occupational therapy in comprehensive care for UHP users. There's a clear trend towards more specialized and evidence-based interventions. The analysis of selected articles reaffirms the existing evidence supporting the diversity and effectiveness of occupational therapy interventions in this context. Moreover, there's a recognized need for interdisciplinary collaboration and active user involvement in their recovery journey. Empirical studies, especially those employing qualitative methods, are predominant. The most common diagnoses addressed in the literature include schizophrenia, depressive disorders, and substance-related disorders. Group interventions are prominent in these settings. The results delineate four categories, emphasizing the pivotal role of occupational therapy in UHP recovery, positively impacting care quality, and fostering performance patterns (meaningful routines). Additionally, the spiritual dimension emerges as significant in the recovery process.
